# Pattern Analysis of Benign and Malignant Atypical Melanocytic Skin Lesions of Palms and Soles: Variations of Dermoscopic Features According to Anatomic Site and Personal Experience

**DOI:** 10.3390/life14060659

**Published:** 2024-05-22

**Authors:** Linda Tognetti, Alessandra Cartocci, Elvira Moscarella, Aimilios Lallas, Emi Dika, Maria Concetta Fargnoli, Caterina Longo, Gianluca Nazzaro, John Paoli, Ignazio Stanganelli, Serena Magi, Francesco Lacarrubba, Paolo Broganelli, Jean-Luc Perrot, Mariano Suppa, Harald Kittler, Roberta Giuffrida, Elisa Cinotti, Lo Conte Sofia, Gennaro Cataldo, Gabriele Cevenini, Pietro Rubegni

**Affiliations:** 1 Dermatology Unit, Department of Medical, Surgical and Neurosciences, University of Siena, 53100 Siena, Italy; alessandra.cartocci@dbm.unisi.it (A.C.); elisa.cinotti@unisi.it (E.C.); sofia.loconte@student.unisi.it (L.C.S.); pietro.rubegni@unisi.it (P.R.); 2 Bioengineering and Biomedical Data Science Lab, Department of Medical Biotechnologies, University of Siena, 53100 Siena, Italy; maurizio.cataldo@gmail.com (G.C.); gabriele.cevenini@dbm.unisi.it (G.C.); 3 Dermatology Unit, University of Campania Luigi Vanvitelli, 81100 Naples, Italy; elvira.moscarella@gmail.com; 4 First Department of Dermatology, Aristotle University, 54124 Thessaloniki, Greece; emlallas@gmail.com; 5 Oncologic Dermatology Unit, IRCCS Azienda Ospedaliero-Universitaria di Bologna, 40138 Bologna, Italy; emi.dika3@unibo.it; 6 Department of Medical and Surgical Sciences (DIMEC), Alma Mater Studiorum University of Bologna, 40138 Bologna, Italy; 7 Dermatology Unit, University of L’Aquila, 67100 L’Aquila, Italy; mariaconcetta.fargnoli@univaq.it; 8 Department of Dermatology, University of Modena and Reggio Emilia, 41125 Modena, Italy; longo.caterina@gmail.com; 9 Skin Cancer Center, Azienda Unità Sanitaria Locale-IRCCS di Reggio Emilia, 42123 Reggio Emilia, Italy; 10 Fondazione IRCCS Ca’ Granda Ospedale Maggiore Policlinico, 20122 Milan, Italy; gianluca.nazzaro@gmail.com; 11 Department of Dermatology and Venereology, Institute of Clinical Sciences, Sahlgrenska Academy, University of Gothenburg, 41390 Gothenburg, Sweden; john.paoli@vgregion.se; 12 Department of Dermatology and Venereology, Region Västra Götaland, Sahlgrenska University Hospital, 41345 Gothenburg, Sweden; 13 Skin Cancer Unit, Scientific Institute of Romagna for the Study of Cancer, IRCCS, IRST, 47014 Meldola, Italy; ignazio.stanganelli@irst.emr.it (I.S.); serena.magi@irst.emr.it (S.M.); 14 Department of Dermatology, University of Parma, 43121 Parma, Italy; 15 Dermatology Clinic, University of Catania, 95123 Catania, Italy; franclacarrubba@gmail.com; 16 Dermatology Unit, University Hospital of Torino, 10126 Torino, Italy; paolobroganelli@gmail.com; 17 Dermatology Unit, University Hospital of St-Etienne, 42270 Saint Etienne, France; j.luc.perrot@chu-st-etienne.fr; 18 Department of Dermatology, Hôpital Erasme, Université Libre de Bruxelles, 1070 Brussels, Belgium; dr.marianosuppa@gmail.com; 19 Department of Dermatology, Institut Jules Bordet, Université Libre de Bruxelles, 1070 Brussels, Belgium; 20 Groupe d’Imagerie Cutanée Non Invasive (GICNI) of the Société Française de Dermatologie (SFD), 75008 Paris, France; 21 Department of Dermatology, Medical University of Vienna, 1090 Vienna, Austria; harald.kittler@meduniwien.ac.at; 22 Department of Clinical and Experimental Medicine, Dermatology, University of Messina, 98122 Messina, Italy; roberta_giuffrida@hotmail.it

**Keywords:** dermoscopy, palmar nevi, palmar melanoma, plantar nevi, plantar melanoma, atypical pigmented palmoplantar lesions, acral melanocytic lesions, acral melanoma, dermoscopic patterns, teledermoscopy

## Abstract

**Background:** The differential diagnosis of atypical melanocytic skin lesions localized on palms and soles represents a diagnostic challenge: indeed, this spectrum encompasses atypical nevi (AN) and early-stage melanomas (EN) displaying overlapping clinical and dermoscopic features. This often generates unnecessary excisions or delayed diagnosis. Investigations to date were mostly carried out in specific populations, focusing either on acrolentiginous melanomas or morphologically typical acquired nevi. **Aims:** To investigate the dermoscopic features of atypical melanocytic palmoplantar skin lesions (aMPPLs) as evaluated by variously skilled dermatologists and assess their concordance; to investigate the variations in dermoscopic appearance according to precise location on palms and soles; to detect the features with the strongest association with malignancy/benignity in each specific site. **Methods:** A dataset of 471 aMPPLs—excised in the suspect of malignancy—was collected from 10 European Centers, including a standardized dermoscopic picture (17×) and lesion/patient metadata. An anatomical classification into 17 subareas was considered, along with an anatomo-functional classification considering pressure/friction, (4 macroareas). A total of 156 participants (95 with less than 5 years of experience in dermoscopy and 61 with ≥than 5 years) from 17 countries performed a blinded tele-dermoscopic pattern analysis over 20 cases through a specifically realized web platform. **Results:** A total of 37,440 dermoscopic evaluations were obtained over 94 (20%) EM and 377 (80%) AN. The areas with the highest density of EM compared to AN were the heel (40.3% EM/aMPPLs) of the sole and the “fingers area” (33%EM/aMPPLs) of the palm, both characterized by intense/chronic traumatism/friction. Globally, the recognition rates of 12 dermoscopic patterns were non statistically different between 95 dermatology residents and 61 specialists: aMPPLs in the plantar arch appeared to be the most “difficult” to diagnose, the *parallel ridge pattern* was poorly recognized and *irregular/regular fibrillar* patterns often misinterpreted. Regarding the aMPPL of the “heel area”, the *parallel furrow pattern* (*p* = 0.014) and *lattice-like pattern* (*p* = 0.001) significantly discriminated benign cases, while *asymmetry of colors* (*p* = 0.002) and *regression structures* (*p* = 0.025) malignant ones. In aMPPLs of the “plantar arch”, the *lattice-like pattern* (*p* = 0.012) was significant for benignity and *asymmetry of structures, asymmetry of colors, regression structures*, or *blue-white veil* for malignancy. In palmar lesions, no data were significant in the discrimination between malignant and benign aMPPLs. **Conclusions:** This study highlights that (i) the pattern analysis of aMPPLs is challenging for both experienced and novice dermoscopists; (ii) the histological distribution varies according to the anatomo-functional classification; and (iii) different dermoscopic patterns are able to discriminate malignant from benign aMPPLs within specific plantar and palmar areas.

## 1. Introduction

Melanoma (MM) is the most aggressive form of skin cancer, responsible for approximately 55,000 deaths per year [1,2]. Among different melanoma subtypes, acral melanoma (AM) is the most frequent in non-white populations, including Asians and Africans, and is responsible for the higher proportion of cases in countries with a lower incidence of melanoma overall [3,4,5,6,7]. AM differs from the other melanoma forms in the biological profile causing specific genetic/immunohistochemical features and related behaviors [8,9,10,11,12]: first, it is a non-UV-related tumor arising from the epithelium-associated melanocytes; second, it shows the early onset of major chromosomal rearrangements with gene copy number changes and multiple high-level amplifications (e.g., driver mutations in GNAQ, NF1, KIT TP53, PTEN, or RB1 genes, versus BRAF and NRAS of superficial spreading and nodular melanoma) [13]; third, it exhibits specific molecular findings (e.g., *CCND1 overexpression*, *AURKA*, and TERT) [14]; fourth, it is characterized by a rapid evolution and ability to metastasize and, thus, a poor prognosis [15,16,17]. This said, AM is also known for having a late diagnosis compared with other forms: the more reported underlying hypothesis emphasize the patients’ (and/or physicians’) reticence in examining this area and the difficulty of the differential diagnosis with acral nevi -with reported rates of misdiagnosis of 20%—despite dermoscopic examination [18,19,20,21]. Dermoscopy is nowadays the most common technique for non-invasive imaging in dermatology, able to provide a “radiography” of pigmented skin lesions through a polarized light source that reaches the dermal–epidermal junction [22]. Many studies were carried out in recent decades aimed at describing and validating a series of dermoscopic criteria helpful in the diagnosis of melanoma arising at different body sites [23,24], clearly showing how the anatomic location condition the dermoscopic features of melanoma on the body [23,24,25], face [26], mucosae [23], and palmoplantar/acral surfaces [27]. Based on this knowledge, corresponding dermoscopic checklists/approaches were derived (e.g., ABCD rules, Menzies method, Chaos and clues, 3-point/7-point checklist) [14]. Importantly, the glabrous skin of palms and soles is anatomically characterized by ridges and furrows and include skin areas subjected to pressure forces of different intensities, ranging from pressure-bearing areas to non-pressure-bearing areas [28]: these two conditions are actually unique compared with other body sites and mirror a peculiar subset of dermoscopic criteria [29,30,31], that require a dedicated training. Moreover, benign melanocytic palmoplantar lesions (MPPLs) have been shown to be “dynamic” as they can exhibit even substantial dermoscopic changes over time despite being histologically confirmed as nevi [32,33]. In addition, acral skin can host a number of atypical melanocytic palmoplantar lesions (aMPPLs) which are clinically and dermoscopically equivocal, and thus, show intermediate histological features of Clark nevi/nevi with mild-to-moderate-to-severe atypia/are classified as ”SAMPUS” (superficial atypical melanocytic proliferation of uncertain significance) lesions [34,35]. Finally, acral congenital nevi represent another dermoscopically difficult entity, characterized by the same dermoscopic criteria found in acral melanoma [36,37]. It is otherwise worth noting that AM is relatively rare in European populations—about 1–2% of all melanoma forms—compared to Asiatic populations [6,7,38]. This may also explain the difficulties encountered by dermatologists in the differential dermoscopic diagnosis with other aMPPLs, which is basically supported by an “educational gap” [18], namely the fact that Caucasian dermatologists are overall less trained on acral aMPPLs than on body melanocytic lesions [16,17,18,19,20,21]. 

A first aim of this study was to submit to dermoscopic pattern analysis of a large series of standardized dermoscopic images of aMPPLs; a second aim was to investigate whether there were recurrent dermoscopic features among aMPPLs located in specific subareas of the palms and soles. 

## 2. Materials and Methods

### 2.1. Study Design

This study was carried out in accordance with the Helsinki Declaration. Approval was obtained by the local ethical committee of Siena University Hospital (*Azienda Ospedaliero-Universitaria Senese, Siena, Italy,* Study Protocol No. 16801) and was then shared with the participating centers. All data were de-identified before use and are kept in accordance with the EU General Data Protection Regulations (GDPR) on the processing of personal data and the protection of privacy in electronic communication (2016/679/EU) [39]. 

The present investigation was carried out as part of the *iDScore-PalmoPlantar* project (*i*-integrated, *D*-dermoscopy, *Score*-scoring system classifiers) focused on the integration of the dermoscopic imaging of the atypical melanocytic lesions of palms and soles with multiple clinical parameters [25,40]. 

The first phase consisted of the development and analysis of an international clinico-dermoscopic database of 542 aMPPLs collected through European countries and hosted on a dedicated web platform (www.iDScore.net, last accessed on 20 May 2024). 

The project was promoted by dermatologists (L.T., P.R.) and technical figures from the Bioengineering and Biomedical Data Science Lab (bioengineer—G.C., biostatisticians—A.C. and S.L.C., data manager/computer expert—G.C.) of Siena University Hospital and proposed to the *Teledermoscopy Working Group* (A.L., M.C.F., I.S., G.N., P.B., J.P., H.K., J.L.P., E.M., F.L., C.L., E.D., M.S., E.C., R.G.) under the Teledermatology Task Force of the European Academy of Dermatology and Venereology (EADV). 

### 2.2. Testing Dataset

A *testing subset* of 471 aMPPLs was derived from the *iDScore-PalmoPlantar* dataset [27] of 542 aMPPLs and was specifically assigned for pattern analysis investigation in a tele-dermoscopic setting. All lesions from the dataset were located on palms/soles and excised for histopathological examination to rule out malignancy. Each case was derived from one patient only. There was no repetition of patients in the dataset. All cases comprised (i) 1 dermoscopic image, (ii) 1 clinical image, and (iii) 3 mandatory lesional data proper to the lesion (i.e., definitive histopathological diagnosis, maximum diameter (mm), precise body location); (iv) mandatory patient data (i.e., sex (F/M) and age (years); (v) patients’ optional data including anamnestic factors (i.e., personal or family history of melanoma (i.e., in a first-degree relative), history of sunburns (>3) in childhood below the age of 14 years, history of labor/sport-related chronic traumatism on the palms/soles) and phenotypic factors (i.e., presence of multiple common nevi [>100] or atypical nevi (AN) [>10] on the body, phototype [I–IV], pheomelanin/red hair phototype, presence of green/light blue/blue eyes, and presence of blond hair) (Table 1). 

Patients were aged at least 18 years, with no limitation in the upper range. The accepted histopathologic diagnoses included nevus with mild/moderate/severe atypia, dysplastic nevus, SAMPUS, melanoma in situ/stage Ia/Ib/IIa (pathologic TNM classification pTis/pT1a/pT1b/pT2a). Additional histopathological data (thickness, mitosis number, regression (%), and presence of lymphocytic infiltrate) were optional.

The 471 cases were provided by 10 European Centers, namely: Siena (Italy), Thessaloniki (Greece), Meldola (Italy), Milan (Italy), Gothenburg (Sweden), L’Aquila (Italy), Turin (Italy), Vienna (Austria), St. Etienne (France), and Naples (Italy)

### 2.3. Anatomic, Functional, and Anatomo-Functional Classifications

**Figure 1:**
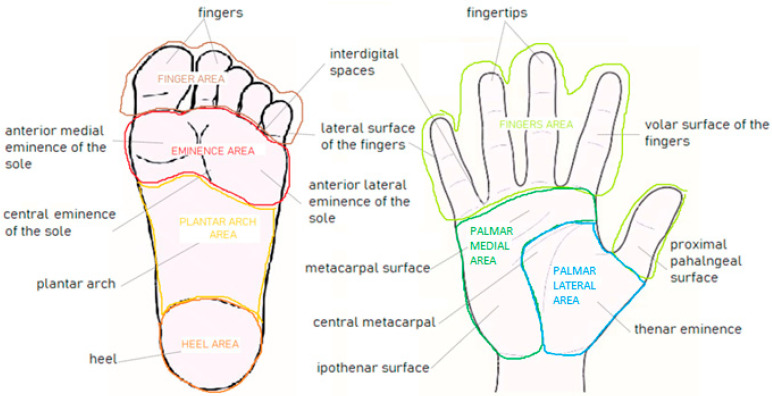
Schematic representation of the anatomic and anatomo-functional classifications used in the testing set of the *iDScore-PalmoPlantar* database, consisting of 17 areas (9 palmar and 8 plantar, *lowercase letters*) and 7 macroareas (4 plantar and 3 palmar, *capital letters*), respectively.

Before the lesion collection start, an “anatomic classification” into 17 subareas, 8 on the soles (i.e., anterior lateral eminence of the sole, anterior medial eminence of the sole, central eminence of the sole, heel, interdigital spaces, lateral surface of the fingers, and plantar region) and 9 on the palms (i.e., plantar surface of the fingers of the sole, central metacarpal, fingertips, interdigital spaces, hypothenar surface, lateral surface of the fingers, metacarpal surface, thenar surface and volar surface of the fingers, and proximal phalangeal surface) was adopted (Figure 1). 

Then, these 17 subareas were grouped according to the intensity of the traumatism/pressure to which each subarea is subjected in a lifetime, and it was estimated according to a “functional classification” into “mild, moderate intense” (Table 2). Finally, based on these two classifications and in order to fulfill statistical purposes, a third “anatomo-functional classification” was adopted, grouping the 17 subareas into 4 macro-areas of the sole (“eminence of the sole area”/”heel area”/”plantar area”/”toe area”) and 3 macro-areas of the palm (“fingers area”/”palmar lateral area”/”palmar medial area”), as illustrated in Figure 1. The average entity of the pressure estimated for each area is indicated in Table 2, where lesion histological distribution is also reported.

### 2.4. Web Platform for Tele-Dermoscopy

Within the web platform dedicated to the *iDScore projects*, the pages reserved to the *palmoplantar lesions* project were available at https://en.idscore.net/projects/palmo-plantar-lesions/palmo-plantar-lesions-2021 (last accessed on 30 April 2024). In particular, all lesions were hosted on the one the registry accessible through the “Site Investigator” menu, linked to the lesion submission section (“Submit image”—(https://docs.google.com/forms/d/e/1FAIpQLSfdvgPwpMbvb4V5J4AfRDSycs0tNMe2AHJsK1ggwFWPsJoY6A/viewform, last accessed on 15 December 2024)). Then, in the section devoted to tele-dermoscopic testing, (https://idscore-pp-testing.web.app/) was created. A large series of blinded tests were performed for both investigational and educational purposes. Each tele-dermoscopic test was personal and it comprised 6 steps, including (i) intuitive diagnosis based on clinical and dermoscopic data + additional patient/lesion data; (ii) pattern analysis; (iii) confidence in diagnosis; (iv) case rating; (v) management) device used. A snowball sampling was performed to enroll participants. Fourteen site investigators (E.M., A.L., E.D., M.C.F., C.L., G.N., J.P., I.S., F.F.C, P.B. JL.P., M.S., R.G., and E.C.) were first invited and they were responsible for the enrolment of at least other 10 dermatologists/dermatology residents who could enroll others. Another 35 participants were recruited by the principal investigator (L.T.) assuming the “Other” (*Site* 0) affiliation. In the end, 156 participants completed the test. 

### 2.5. Participants’ Data

The test could be performed by either dermatologists or dermatology residents/plastic surgeons. The only requirement was to indicate, before starting the test, their age, and sex, their affiliation center (site 0–14), their education country, the preferred device for testing, and declare the degree of experience in dermoscopy as follows: *level I*—less than 1 year of experience; *level II*—1–4 years of experience; *level III*—5–8 years of experience; *level IV*—more than 8 years of experience. This skill classification took into account the training time for dermatologists across Europe and was successfully employed in all previous *iDScore* studies [24,25,26,40]. The system of site affiliation ensured that all participants received only cases provided by other centers in their personal test, as previously validated [24,25,26,40].

### 2.6. Tele-Dermoscopic Pattern Analysis

**Figure 2:**
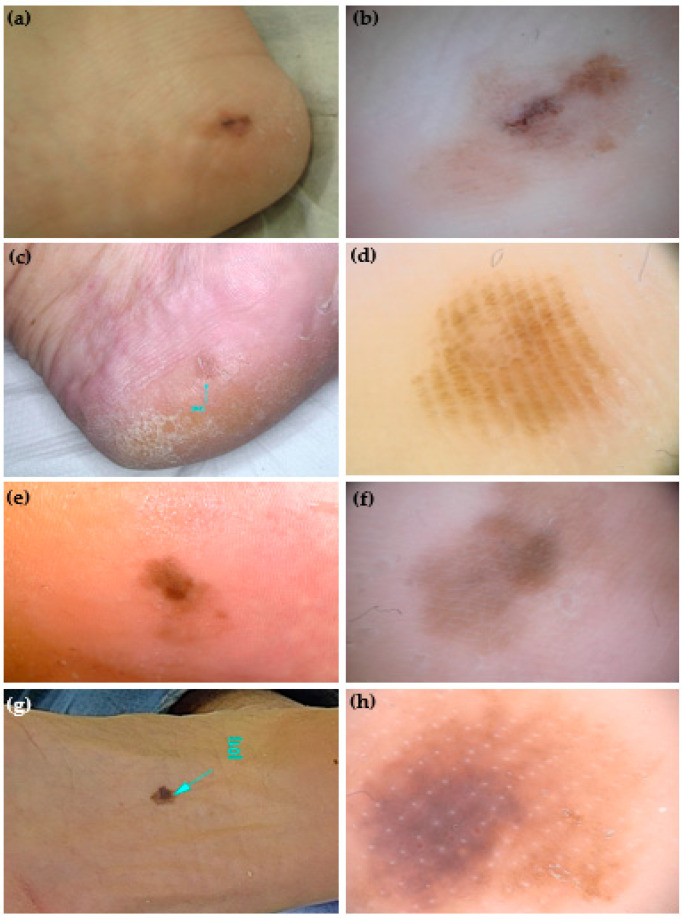
Clinical and dermoscopic (polarized light, 20×) appearance of 4 atypical melanocytic palmoplantar lesions (aMPPLs) of the sole, localized at the heel (**a**,**c**) and plantar region (**e**,**g**). Both lesions of the heel (**a**–**d**) belong to women aged 44, have a similar appearance in terms of a diffuse brownish pigmentation and diameters (10 and 8 mm, respectively), but different dermoscopic patterns: asymmetry of structures and colors, irregular blotches are visible in the melanoma (**b**), while homogeneous pigmentation arranged in a regular fibrillar pattern in a nevus (**d**). Both lesions of the plantar region (**e**–**h**) were seen in males aged 70, had a similar appearance of a brownish multicolored macules and similar diameters (10 and 11 mm, respectively) but differed in dermoscopic aspects. The asymmetry of structures and colors, and irregular diffuse pigmentation (**f**) in a melanoma, where, in the loaded area, the eccrine pores appear as white lines within an irregular blotch; in the non-loaded area, a parallel-ridge pattern is visible. In the acral nevus, a non-typical pattern with a focal pigmentation is seen along with regularly distributed eccrine pores (white dots) (**h**).

**Figure 3:**
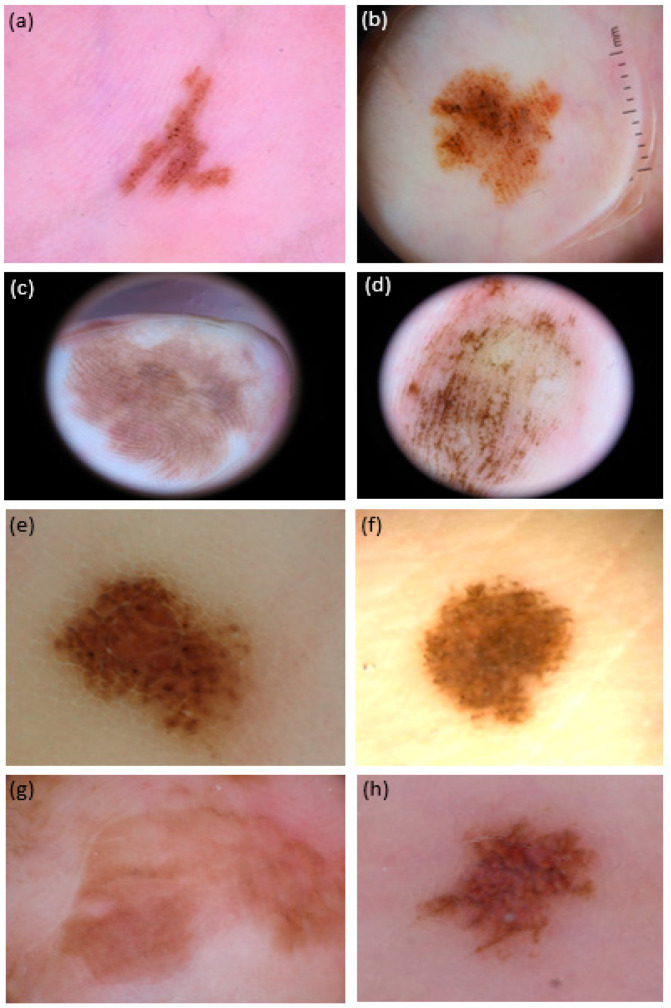
Dermoscopic pictures (polarized light, 20×) of 8 atypical melanocytic lesions (aMPPLs) localized in different areas of the palm, either pressure-bearing area (i.e., ipothenar eminence: (**a**,**b**), and fingertips; (**c**) volar surface of the fingers; (**d**) lateral surface of the fingers; (**e**) thenar surface; (**f**) and no pressure-bearing (i.e., central metacarpal (**g**,**h**)). Both lesions on the thenar eminence (i.e., pressure-bearing area) were seen in females and show the parallel-furrow + globular pattern: however, lesion (**b**) also exhibited asymmetry of colors and irregular blotches, was 11 mm in diameter and belonged to a 70-year-old patient: the histology report was consistent with acrolentiginous melanoma; of converse, lesion (**a**) was 5 in diameter and belonged to a 30-year-old patient—the histology was in favor of a congenital nevus. Lesions on the fingers were both 20 mm in diameter and showed the asymmetry of structures: however, lesion (**c**) also showed a clear parallel-ridge pattern and was observed in a 62-year-old female (histology: melanoma pTis), while lesion (**d**) showed basically a parallel-furrow-pattern and belonged to a 19-year-old female (histology: congenital nevus). Both lesions at the thenar eminence/proximal phalangeal surface of the thumb (**e**,**f**) showed a diameter of 5 mm and a globular pattern, but eccrine ducts hostia were visible as white dots only in lesion (**f**), exhibited by a 33-year-old female and consistent with a congenital nevus; lesion (**e**) was otherwise interpreted as a borderline lesion (nevus with severe atypia/SAMPUS) in a 41-year-old male. Lesions on the central metacarpal area of the hand, no subjected to chronic pressure, appeared to have reticular pattern with brownish pigmentation under dermoscopy: lesion (**g**) was 25 mm in diameter, belonged to a 88-year-old female and was an in situ MM (pTis) while lesion (**h**), although exhibiting multiple colors (reddish and brownish areas) was actually a nevus of 5 mm in a 45-year-old-female.

Each teledermoscopic test was carried out over 20 blinded cases. The participant should assess the presence/absence of a panel of 12 dermoscopic patterns (Figure 2 and Figure 3). This list includes dermoscopic structures that are the most frequently reported in studies, suggesting either malignancy or benignity, based on literature data: *asymmetry of structures, asymmetry of colors, parallel ridge pattern, irregular blotches, regression structures, blue-white veil, irregular streaks, irregular diffuse pigmentation, irregular fibrillar pattern, parallel furrow pattern, regular fibrillar pattern, lattice-like pattern* [4,7,14,23,29,30,31,32,33,36,37,38,41,42,43,44]. In order to facilitate the comprehension of a given dermoscopic criterion, a link was accessible in each test page reporting both the established dermoscopic criteria description and an exemplificative dermoscopic picture. This was of significant help to all novice participants in recognizing the required pattern in the tested case. Each test could be suspended and restarted at any time and rested available for a 4-month interval. 

### 2.7. Statistical Analysis

Images were evaluated from a minimum of 2 to a maximum of 12 times, and a mean of 7 times (the total number was 3120). The presence of each feature was assessed according to most readers. Descriptive statistics was carried out. Absolute frequencies and percentages were estimated for qualitative variables, while mean and standard deviation for the quantitative ones. Student *t* test was performed to compare age and diameters among histopathologically confirmed diagnostic groups. The chi-squared test or *Fisher* exact test was performed to evaluate the association between the features assessment and diagnosis and specific palmo-plantar location. A *p* value < 0.05 was considered statistically significant. Analyses were carried out with R version 4.3.1.

## 3. Results

### 3.1. Tele-Dermoscopic Tests

A total of 156 tests were completed by 156 participants from 17 countries, aged on average 35.1 ± 10.0, 77 (49.4%) males and 79 (50,6%) females. According to their level of experience in dermoscopy practice, participants were grouped as follows: 30 (19.2%) of *skill level I*, 65 (41.7%) of *skill level II,* 24 (15.4%) of *skill level III,* and 37 (23.7%) of *skill level IV.* Thus, participants of *skill level I + II* were conventionally named “novices” and corresponded to 95 dermatology residents, while participants of *skill level III + IV* were named “experts” and corresponded to 61 dermatologists. The indicated preferred device for testing was the personal computer for 64 participants (41%), the smartphone for 45 participants (28.8%), the notebook for 20 participants (12.8%), and another for 1 participant (0.6%); some indicated two device contemporaries, e.g., smartphone + personal computer –(n = 11, (7.1%), personal computer + notebook –(n = −1, (0.6%), personal computer + smartphone –(n = 3, (1.9%), smartphone + notebook –(n = 4, (2.6%). Three devices were indicated as preferred by a few participants (n = 5).

### 3.2. Case Study Characteristics 

The collected data concerning patients and lesions characteristics/demographics of 471 aMPPLs cases are summarized in Table 1. Among them, 94 (20%) were early melanomas (EMs) and 377 (80%) were ANs. This ratio was the one required to have a dataset adequately balanced for statistical purposes. The sex of the patients was not homogeneously distributed: indeed, 65% (307) of all aMPPLs cases belonged to women, and among them, 84% was represented by AN and only 16% by EM, those difference was statistically significant. Of converse, EM cases in men were 27.4% of all male cases. Concerning age, the average value for all patients was 45.39 ± 18.96 years, with melanoma patients being significantly older (63.97 years on average). The diameter was 8.24 ± 6.3 mm on average and was significantly different (<0.001) between AN—6.49 mm—and EM—15.30 mm. Patients were prevalently of phototype III (52% of patients); no significant data were obtained from the remaining anamnestic investigations.

### 3.3. Anatomic Distribution of aMPPLs 

Cases were distributed through feet and hand surfaces, with a clear prevalence for the soles, i.e., 435 cases (92,4%) (Table 2). With regard to the anatomic distribution, the subarea (among the 17 of soles and palms) that hosted the majority of aMPPLs cases overall was the “plantar arch” area (207 cases, 28% EM and 47% AN). Concerning the malignant aMPPLs, the area with the highest number of EM overall was the “plantar region” (27 cases) followed by the “heel” (23 cases). However, the area hosting the highest rate of EM out of AN was the ”heel” area, with EM accounting for 40.3% of aMPPLs cases at this site. Then, the area hosting the major rate of EM out of AN of the hand was the “metacarpal surface” (33.3% of aMPPLs cases at this subsite), followed by “thenar surface” (22%). In addition, the distribution analysis of EM according to eight subareas of the sole demonstrated a significant prevalence on the heel site (*p* < 0.001), while no significant data were detected on the palm.

Regarding AN, the most involved area at all was the plantar arch (180 cases), with no significant differences in the numerosity of the other soles area (range of 18–34 cases). Nevi of the hand were globally homogeneously distributed, ranging from eight cases of the “hypothenar surface” to 0 of the “fingertips”. The distribution analysis of AN according to eight subareas of the sole demonstrated a significant prevalence of on the plantar arch site (*p* = 0.001), while no significant data were detected on the palm.

### 3.4. Anatomo-Functional Distribution of aMPPLs

The comparison of a functional classification of the 17 subareas with the anatomic one, produced no conclusive results in terms of correlation between the traumatism/pressure and number of aMPPLs. The same can be said for the AN, as the highest quote was indeed present in a no-pressure area (plantar arch), reaching statistical significance (*p* = 0.001). Of interest, we observed a high relative incidence of MM in the areas subjected to intense/chronic pressure such as the “heel” and the “anterior medial eminence”, or the “metacarpal surface” and “lateral surface of the fingers” (Table 2). According to the macro-area distribution analysis, EMs were statistically prevalent on the heel area (*p* < 0.001). No significant distribution data were derived from palmar macroareas.

### 3.5. Pattern and Anatomic Distribution 

A total of 37,440 dermoscopic data were obtained from the tele-dermoscopic 3120 pattern evaluations of 12 patterns, reaching an average number of 7 evaluations per image.

#### 3.5.1. Variation of Dermoscopic Features through Plantar Areas

Table 3 illustrates the results of the pattern analysis carried out by 148 participants variously skilled in tele-dermoscopy, namely dermatology residents (skill levels I–II) and dermatologists (skill levels III–IV), on both benign and malignant aMPPLs located on the soles. For each of the 12 dermoscopic patterns, the entity of the variation through the subareas was also statistically investigated. 

According to the evaluations of 87 dermatology residents in 86 plantar melanoma cases, the most frequently reported patterns overall were the *asymmetry of colors* and *asymmetry of structure,* followed by *regression structures* and *blue-white veil*: this trend was shared by all plantar areas. Of note, the *parallel furrow pattern* and the *regular fibrillar* and the *lattice-like pattern* were also identified in the eminence of the sole area, where the *irregular fibrillar pattern* was not detected. Then, the *parallel furrow pattern* was not recognized in the *toe* and plantar arch areas. Interestingly, the *parallel-ridge pattern* was identified in a few cases. The variation of each one of the 12 dermoscopic patterns through the four plantar areas were not statistically significant (*p >* 0.05) (Table 3)

According to the evaluation of AN cases, different dermoscopic descriptions appeared among the subareas. The “plantar arch” was the more challenging area for residents, as both benignity and malignancy-suggestive features were detected: namely, the *parallel furrow pattern,* the *regular fibrillar pattern,* and the *lattice-like pattern* were recognized in 14–27% of cases, as well as *asymmetry of colors* and *asymmetry of structures* in 29–24% of cases. A similar trend was observed in the AN of the “toe area” (*parallel furrow pattern* in 30% of cases and *asymmetry of colors* and *asymmetry of structures* in 27–29% of cases) and in the eminence of the “sole area” *(parallel furrow pattern* in 21% of cases versus *asymmetry of colors* and *asymmetry of structures* in 30–27% of cases). Only in AN on the “heel area” was there a slight prevalence of benignity-suggestive features (i.e., *lattice-like pattern*) observed. More specifically, three features appeared to vary significantly (*p* < 0.05) among plantar areas in AN cases, which were considered individually: the *irregular streaks* predominate on the AN of the eminence of the sole and toe; both the *irregular fibrillar pattern* and the *regular fibrillar pattern* were prevalent on the plantar arch (*p* = 0.024). In 314 AN cases evaluated by dermatologists (skill levels III–IV), only two benign-like features were significantly recognized in specific areas, namely the *parallel furrow pattern* in the “toe area” (38% of cases) and the *lattice-like pattern* in the “heel area” (42%). Then, two malignancy-suggestive patterns, the *asymmetry of colors* and *of structures,* were still prevalent at “toe area” (32% of cases).

#### 3.5.2. Variation of Dermoscopic Features through Palmar Areas

Table 4 reports the results of the pattern analysis carried out by 56 participants variously skilled in tele-dermoscopy, namely dermatology residents (skill levels I–II) and dermatologists (skill levels III–IV), on both benign and malignant aMPPLs located on the palms. For each of the 12 dermoscopic patterns, the entity of the variation through the subareas was also statistically investigated.

- Skill-levels I–II

According to the evaluations of 26 dermatology residents performed in 28 palmar AN cases, the predominant features were the *asymmetry of colors* and *asymmetry of structures* at the palmar medial and lateral areas. The other features were poorly recognized in both AN and in eight EM cases.

- Skill-levels III–IV

Examining the evaluations of 30 dermatologists performed in 26 palmar AN cases, the predominant feature was the *parallel furrow pattern* in both the palmar lateral (58%) and medial (50% of cases) areas. Of note, the *asymmetry of colors* pattern was recognized in 5 out of 12 palmar lateral AN.

In eight EM cases, the *parallel ridge pattern* and the *asymmetry of colors* were described in 3 out of 8 cases.

### 3.6. Dermoscopic Features and Anatomo-Functional Distribution

#### 3.6.1. Distribution Analysis of Dermoscopic Patterns According to Histology and Plantar Location

Table 5 summarizes the analysis of frequency distribution between 12 dermoscopic features and specific anatomo-functional macro-areas of the soles, according to experts’ pattern analysis, aimed to investigate a series of the possible association of patterns within 400 malignant/benign aMPPLs. For this investigation, only dermatologists’ evaluations were taken into account, in order to avoid experience-related bias. Globally, two dermoscopic patterns were statistically different in distinguishing malignant from benign aMPPLs in all plantar areas, i.e., the *asymmetry of colors* and *the regression structures.* Other malignancy-suggestive features were detected in two areas, e.g., the *blue-white veil* and the *irregular blotches,* in a single area only (i.e., the *irregular diffuse pigmentation* in the plantar arch according to residents’ evaluations). No benignity-suggestive features appeared significant from the univariate analysis.

- Eminence of the sole

This area includes the three subareas of anterior eminence; central eminence; and antero-medial eminence, which are characterized by intense pressure. According to experts’ pattern analysis, the most recognized feature in EM cases was the *asymmetry of colors* (64.7%) and in AN cases was the *parallel furrow pattern* (40%).

Concerning the malign/benign comparison, three dermoscopic patterns appeared to be more represented in EM (17 cases) than in AN (50 cases) with a statistically significant difference (*p* < 0.05), namely *asymmetry of colors, regression structures,* and *blue-white veil.* Of note, *the parallel ridge pattern* was described more frequently in AN (7 out of 50) than in MM cases (2 out of 17). None of the three benignity-suggestive features (i.e., *parallel furrow pattern, regular fibrillar pattern, lattice-like pattern*) was found to statistically discriminate benign from malignant aMPPLs, but the most described pattern overall was the *parallel furrow pattern* in the AN cases according to descriptive analysis.

- Toe

The toe area, which includes the plantar surface of the fingers, the lateral surface of the fingers, and the interdigital spaces, can be regarded as a macro-area undergoing moderate ad variable trauma. According to expert evaluations, the *asymmetry of structures* was detected in 95% of EM cases and the *parallel furrow pattern* in 38.2% of AN cases. 

According to univariate association analysis, five dermoscopic features were significantly more recognized in EM cases (19) than in AN cases (68), such as the *asymmetry of structures, asymmetry of colors, irregular blotches, regression structures,* and *irregular diffuse pigmentation.*

- Plantar arch

The pattern analysis of the plantar arch, which is regarded as a non-pressure bearing area, produced several significant data. Among EM cases, the most frequently identified pattern was the *asymmetry of colors* (85.2%), followed by the *asymmetry of structures* (81.5%); among AN cases, the lattice-like pattern predominates (28.5%) but the *irregular diffuse pigmentation* was prevalent compared with other areas according to residents’ evaluations. 

The malignancy-suggestive features that differ significantly in 27 EM compared to 165 AN, were the *asymmetry of structures, asymmetry of colors, regression structures,* and *blue-white veil.* Of converse, the benignity-suggestive features discriminating AN were the *parallel furrow pattern* (*p* = 0.013) and the *lattice-like pattern* (*p* = 0.012).

#### 3.6.2. Distribution Analysis of Dermoscopic Patterns according to Histology and Palmar Location

The results of the distribution analysis of 12 dermoscopic features among specific anatomo-functional macro-areas of the palms according to the benign/malignant histologic classification of 34 aMPPLs is reported in Table 6. In order to avoid experience-related bias, only dermatologists’ evaluations were considered. Due to the low numerosity of the subgroups, the association analysis produced no significant results. According to descriptive analysis, we observed that the *parallel furrow pattern* was the only pattern to be more frequently recognized in AN than in EM cases, at all sites and particularly at palmar lateral area (intense traumatism). Of note, none of the malignancy-related features were more frequent in EM than in AN: in particular, the *parallel ridge pattern* was detected in 4 out of 6 AN cases and the *asymmetry of colors* in 5 out of 12 AN cases.

## 4. Discussion

The differential diagnosis of benign and malignant MPPLs is not always easy, even with the dermatoscope, and thus, they are defined as melanocytic proliferation at “special site” [23,33,41,42,43,44]. It is possible to affirm that this is a challenging diagnosis, indirectly, based on two data: first, the late diagnosis of palmoplantar melanomas and the high misdiagnosis rate [20,21]; second, the high number of excisions/biopsies of benign nevi on soles and palms [18,30,38,42]. Unfortunately, we cannot affirm this directly, at least in a Caucasian population, as large prospective studies on the dermatologists’/residents’ perception and/or on their experience on those lesions is still lacking. However, it was recently stressed by Costello et al. how American dermatologists may have both education-related and practice-related gaps (i.e., poor accuracy in the recognition of low-risk benign lesions and of specific acral dermoscopic pattern, as well as poor patient compliance with follow-up) [18]. Indeed, melanocytic palmoplantar lesions are particularly risky: on one hand, the acral melanoma has a poor prognosis due to the intrinsic speed of growth and a high metastatic potential [4,5]; on the other, a surgical incision/excision on palmar/plantar skin area brings both functional and aesthetic consequences for the patients, which should be considered in case of histologically benign lesions [18]. 

To date, aMPPLs have been poorly investigated in Caucasians populations compared to Asiatic populations and the hypothesis that the dermoscopic knowledge derived from Asiatic studies may be not fully applicable to Caucasians lesions was raised [18,45]. First, the palmar and plantar sites, which are actually distinct, were usually merged and studied together [41,42,43,44,45,46,47,48]. Second, studies were specifically focused either on inconspicuous benign-looking acral nevi (with a diagnosis mainly estimated by consensus and follow-up, in the absence of a histologic definitive histopathologic diagnosis) [29,30,31,32,36,37,38,41,48,49,50,51] or on acral melanomas [42,43,44,47]. Third, the anatomic distribution of MPPLs was rarely investigated and, in those cases, most exclusively in Eastern Asiatic and/Turkish populations, on foot plantar lesions in monocentric case studies [41,52,53,54,55,56]. In parallel, it seems that the knowledge regarding the peculiar “acral dermoscopic glossary” [28] is not as comprehensive as the dermoscopic glossary for body or facial lesions, being almost inadequate to cover the spectrum of aMPPLs [18,19,20,21,33,41,42,43,44,45,46,47,48,49]. 

Thus, there was a need to perform a detailed dermoscopic analysis on a large series of aMPPLs in Caucasian patients. 

We recently demonstrated that setting up large integrated clinic-dermoscopic datasets collecting equivocal melanocytic lesions of the body (*iDScore-body* dataset) [50] and of the face (*iDScore-facial* dataset) [51] could beneficially increasing knowledge about anatomical correlations and improved dermatologists’ accuracy. Moreover, our group previously showed that a deep learning provisional model (e.g., digital dermoscopy analysis) derived from an integrated dataset could support the differential diagnosis of acral lentiginous melanoma in situ from acral junctional nevus [33,49] 

The present study analyzed, for the first time, a series 471aMPPLs excised from Caucasian patients, from a clinical and dermoscopic point of view. It appears innovative from the following aspects:

(i) The *anatomical classification* into 18 subareas adopted here was the most detailed used so far [52,53,54,55,56,57], since previous studies took into account only 2 or 3 areas of the sole area, based either on the anatomy [41,52,53,54] or on the functional criteria [56].
(ii) The *anatomo-functional* classification derived on this basis was also new as no cross-analysis was previously carried out between the anatomic location of MPPLs and their pattern analysis according to the pressure/traumatism/friction entity and according to the histopathology. [51,52,53,54,55] Here, we found the significant association of EM cases distribution on the macroareas “heel” (40.3% of cases, *p* < 0.0001) and “eminence of the sole” area (34% of cases). Of note, these two macroareas also have the highest degree of friction/traumatism and pressure among the sole areas. These distribution trends are in line with Japanese [54,57], Korean [58], and U.S. [59] monocentric studies on melanoma distribution on the soles concerning a predominance of the heel site, which can be considered the weight-bearing area subjected to major pressure/cm^2^.
(iii) The majority of AN cases were distributed on the “plantar area” (87% of cases), followed by the toe area (“interdigital spaces” + “lateral surface of the fingers” + “plantar surface of the fingers” subareas). Of note, the “plantar area” and the “toe area” were subjected to no and mild pressure, respectively. Instead, in a Japanese population study by Miyazaki et al., the quote of acral nevi (n = 298) was similar among non-pressure-bearing (“plantar arch”) and pressure-bearing areas (eminence of the “sole” + ”heel” areas). [56] To date, only one study by Ghanavatian et al. took into account atypical nevi (25) beside typical ones (137), but cases were classified by histopathology and not by dermoscopy, and globally produced similar results in the comparison with 73 EM cases [54]. Similar literature data were reported in ethnic studies on benign-looking acral nevi in Mexican [41] and Korean [53] populations, where the plantar arch hosted the majority of acquired nevi cases.
(iv) Concerning the anatomic and anatomo-functional distribution of aMPPLs on the palms, the highest relative quote of EM/aMPPLs was found in the “fingers area” (33%), mainly due to cases on the fingertips, where traumatism/friction is intense. Conversely, the subarea hosting the majority of AN was the “hypothenar surface”, corresponding to a site of moderate traumatism. These data are otherwise in line with the unique study investigating the anatomic density of melanomas and clear-cute benign acquired acral nevi on the palms, carried out on 34 Japanese patients [55]. The present anatomo-functional classification seems to confirm the hypothesis of a causative role in chronic traumatism/friction and mechanical stress in eliciting the malignant clone proliferation in predisposed subjects that was only raised based on monocentric studies but never confirmed on a large dataset [54,57,58,59,60]. Intense/unique traumatism was only reported anecdotally as inducing nodular melanoma at palms [61,62] but further data are needed, and the current knowledge is biased because of the fact that palmar melanoma is relatively rare in Caucasians. In addition, a melanomatous proliferation borderline with in situ acrolentiginous melanoma was recently described in a non-bearing area such as the plantar arch, induced by a unique curettage-related trauma [61]. Nevertheless, the investigation of anamnestic data concerning the “*Chronic traumatism of palms*” and the *“Chronic traumatism of soles”* performed during case collection produced poor data, which was inadequate for performing detailed statistical analysis. This can be attributed to the fact that most dermatologists usually do not collect/write down this kind of data in clinical reports during mole check visits [27].
(v) The tele-dermoscopic setting allowed a huge number of dermatologists (61) and dermatology residents (95), performing pattern analysis over a series of 471 aMPPLs corresponding to the *testing subset* of the *iDscore palmoplantar dataset* of 542 cases [27]. In the pattern analysis of 86 dermoscopic images of EM cases of the sole, the evaluations of dermatologists’ and residents were not significantly different. The overall highest degree of concordance between the residents and dermatologists was obtained from the assessment of the parameters *asymmetry of structures*, followed by *asymmetry of colors*, considering all subareas.
(vi) Notably, a series of dermoscopic findings appeared to be new compared with previous literature, essentially based on clear-cut lesions in different populations, as mentioned previously.

First, the recognition of the *parallel furrow pattern*, which was traditionally considered the easiest/more didactic, was highly variable both among the two participants groups and among solar subareas. Moreover, the fact that the *parallel furrow pattern* was detected by experts in eight EM cases (four on the “eminence of the sole” and four on the “toe” area, statistically significant variation) can stimulate some hypotheses: first, the *parallel furrow pattern* may be under-recognized in AN, due to the combination with other patterns, or misdiagnosed with an irregular *fibrillar pattern* in EM cases, while it is easily recognized as clear-cut acquired acral nevi [18,30,44,63]. Furthermore, the *parallel furrow pattern* was not the prevalent feature recognized in the AN of the plantar arch, differently from previous studies [30,52].

Secondly, both the *irregular and regular fibrillar patterns* were significantly more recognized on the plantar arch than in other areas: these data are new compared with the few literature data where the *fibrillar pattern* is reported in weight-bearing areas and the *irregular fibrillar* pattern is reported in transitional areas beside the Wallace line and in some weight-bearing areas [52,53,54,55,56]. Moreover, recent studies confirm that many *regular fibrillar* patterns observed in weight-bearing areas actually result from an “optical transformation” of *parallel furrow patterns* [64].

Third, the *parallel ridge pattern* was poorly recognized by both experts and residents (17 out of 86 EMs for experts, 14 out of 86 EMs for residents): a recognition-bias is less likely to be causative, while the specific dermoscopic appearance of aMPPLs in Caucasian people may be the main reason to address. Of note, the highest number of EMs exhibiting the *parallel ridge pattern* (8) were found on the heel.

(vii) Taking into account the pattern analysis data derived from experts evaluations, two features appeared to vary significantly (*p* < 0.05) among EM cases (*asymmetry of structures* predominates on the “toe” versus other three areas, *parallel furrow pattern* on the “toe + eminence of the sole” compared with the other areas) and two features among AN cases (*p* < 0.05): the *parallel furrow pattern* on the “eminence of the sole” + “toe” versus other areas; the *lattice-like pattern* on the “plantar arch” + “heel” versus other areas.
(viii) It should also be noted that, in AN cases of the plantar area and toe, both residents and experts more frequently recognized two malignancy-suggestive patterns (*asymmetry of colors* and *asymmetry of structures)*, than benignity-suggestive patterns such as *parallel furrow pattern, regular fibrillar pattern*.
(ix) Concerning palmar aMPPLs, there were no significant differences in pattern recognition as performed by residents or dermatologists, in both EM and AN cases. Since the group of aMPPLs of the palms was not large, only descriptive analysis can be considered and data concerning the concordance analysis between dermatologists’ and residents cannot be performed. Globally, the “palmar medial area” composed of the “hypothenar surface” and “metacarpal surface”, subjected to moderate pressure, hosted the highest rate of aMPPLs. Among them, either challenging AN cases (that is, lesions exhibiting an *asymmetry of structures* and *asymmetry of colors)* and AN with *parallel furrow pattern.* Differently, in the lesions on the ”fingers area”, it was easier to recognize the benignity-suggestive pattern in AN cases and the malignancy-suggestive pattern in EM cases. These data may suggest that a degree of pattern interpretability/dermoscopic difficulty of aMPPLs of the palmar area is lower than the aMPPLs of the finger area [52,55].
(x) The cross-analysis of dermoscopic pattern distribution through soles/palms subareas according to histologic output produced interesting results. In palms, where subgroups’ numerosity was very reduced, the data from descriptive analysis suggests that the *parallel furrow pattern* was easily recognized in all cases, independently from histologic outcome, and particularly at palmar medial area. On the other hand, AN cases were actually equivocal, having the *parallel ridge pattern* in 4/6 and the *asymmetry of colors* in 5/12 AN cases. We found two dermoscopic patterns able to statistically discriminate malignant from benign aMPPLs located in any area of the sole, namely *asymmetry of colors* and *regression structures*. Previous analysis also demonstrated that these two criteria were easily recognized by both groups of participants in malignant cases, and were often described in challenging AN cases, such as those on the plantar arch. No pattern was highly specific for benignity in all cases.

In line with the findings from descriptive analysis, the *parallel ridge pattern* was not found to be significantly associated with malignant cases. Since the management algorithms available to date indicates that the *parallel ridge pattern* is the most important feature to discriminate malignancy of acral lesions, as derived by Japanese/Korean population studies [44,46,63,64,65] carried out on clear-cut MPPLs cases, there was the need for studies to compare the Asiatic appearance of malignant cases with European aMPPLs. This limit in the management strategy was otherwise previously highlighted by Costello et al., suggesting the introduction of the multi-component pattern in the 3-step algorithm in order not to miss EMs up to 6 mm without parallel ridge pattern [18]. Then, considering each sole area, detailed significant associations were revealed. In summary:

₋ The *toe area* (*interdigital spaces + lateral surface fingers + plantar surface fingers*), subjected to none-to-moderate pressure/traumatism, was characterized by the easiest-to-diagnose EM cases, having the highest number of discriminant malignant features, i.e., *asymmetry of structures, asymmetry of colors, irregular blotches, regression structures, and irregular diffuse pigmentation*. Conversely, AN cases in this area were moderately difficult-to-diagnose, having similar rates of benign and malignant features.
₋ In the *heel* area (subjected to the major pressure/cm^2^), the *parallel furrow pattern* and the *lattice-like* patterns were able to statistically differentiate benign from malignant aMPPLs (*p* = 0.014 and *p* = 0.001, respectively). In parallel, two patterns discriminated malign from benign aMPPLs, the *asymmetry of colors* (*p* = 0.002) and the *regression structures* (*p* = 0.025). This suggests that the differential diagnosis according to pattern analysis between AMs and EMs is somehow easiest in the *heel area*, where there are two malignant and two benign features available: this may be ascribed both to the highest number of melanoma cases overall and to the anatomical structure of heel skin undergoing intense/chronic pressure that determines a more polarized profile than other aMPPLs cases.
₋ In the *eminence of the sole* area (*anterior lateral eminence* + *anterior medial eminence + central eminence,* subjected to moderate–intense pressure, Figure 1), three patterns statistically discriminated malignant from benign aMPPLs, namely *asymmetry of colors* (*p* = 0.025), *regression structures* (*p* = 0.003), and *blue-white veil* (*p* = 0.001). On the other hand, the features suggestive of benignity were not specific, with the *parallel-furrow pattern* recognized in 40% of AN cases versus 23.5% EM cases. Thus, this area can be characterized as a moderately difficult area.
₋ In the *plantar arch*, the *lattice-like pattern* was statistically significant in distinguishing benign cases (*p* = 0.012): this trend was specific for this plantar area, where AN cases turned out to be particularly equivocal/difficult-to-describe. Moreover, four discriminant malignant features (*p* < 0.05) were detected (*asymmetry of structures, asymmetry of colors, regression structures,* and *blue-white veil)* but these data can be determined by the high number of AN cases (165) compared to EM cases (27) in this area.

This study has some limitations. First, the number of palmar cases was low, especially the malignant quote. This trend was not dependent on the case selection strategy but rather reflects the epidemiological situation of palmar melanoma in European countries, where this entity is extremely rare [27] (nail melanoma was not included in the study for different dermoscopic appearance). Second, the present dataset was derived from a “filtered population” and may be regarded as a real difficult subset of cases: indeed, as the first level of lesion selection was carried out by skilled dermatologists that took the decision to excise the lesion, the second level of selection was retrospectively performed by the 10 *site investigators* among the cases excised in their Skin Cancer Screening Center, and the third level of selection was performed by the PI. A third point to be underlined is that clear-cut congenital lesions below the age of 19 were excluded in order to avoid potential bias in comparative dermoscopic pattern analysis: that congenital nevi may exhibit ugly features (*parallel ridge pattern, irregular diffuse pigmentation, asymmetry of structures and colors*—i.e., multicomponent pattern) especially in children/adolescents, but the anamnestic data of patient age drives the correct management. Consequently, we decided not to include the specific dermoscopic patterns described for acral congenital nevi, such as *Globular pattern + parallel furrow pattern* (“*peas in a pods*”) [36,37] for pattern analysis. Finally, we decided to limit the list of required patterns to analyze in teledermoscopy to 12 items, in order to ensure the feasibility of the whole test, reaching a balance between the length of the procedure and the time required online (i.e., each of the 20 lesions was tested through a series of six consecutive phases, namely *diagnosis, pattern analysis, case rating, confidence in diagnosis, management, device*) [27]. For this reason, and considering the dermoscopic difficulty of the dataset, the pattern items were selected favoring the number of malignancy-suggestive features compared to benignity-suggestive features; consequently, some patterns recently described in a monocentric study—the *homogenous pattern* and the *reticular pattern*—[30] were not included. 

## 5. Conclusions

The differential non-invasive diagnosis of aMPPLs remains challenging due to the so-called “biological overlap”. The traditional risk factors addressed for body melanoma appear not to be helpful. The present dataset of palmoplantar difficult lesions is currently the larger and more detailed dataset available for European patients with aMPPLs. In line with literature data, the areas with the highest density of EMs compared to ANs were the heel (40.3% EM/aMPPLs) for the sole and the “fingers area” (33%EM/aMPPLs) for the palm: both sites are characterized by intense/chronic traumatism/friction.

Concerning pattern analysis, the recognition rates of 12 dermoscopic patterns were not statistically different between residents and dermatologists. Thus, a series of consideration can be derived:

- The group of AN cases collected herein actually exhibited equivocal features and this was independent from the examiners’ personal experience in dermoscopy
- The *asymmetry of colors* and *regression structures* turned out to be statistically significant in the differentiation of EMs from AN, in any area of the sole, suggesting that these two dermoscopic patterns are more specific because they are more easily recognizable in EM than in AN, whereas the *parallel ridge pattern* appears as an equivocally interpreted feature.
- The *parallel ridge pattern* was globally poorly recognized in EM cases at all plantar sites compared with studies on Asiatic populations [52,53,54,55,56].
- The parallel furrow pattern was globally poorly recognized in AN cases, probably due to a combination with another pattern.
- There was is an objective difficulty in discriminating the *irregular fibrillar pattern* from the *regular fibrillar pattern* among aMPPLs, both in dermatologists and residents’ evaluation, in line with recent reports [18,64] (nevertheless, the discrimination between *regular* and *irregular fibrillar pattern* and between parallel *furrow pattern* appearing as a *regular fibrillar* in weight-bearing areas is still a matter of debate) [64]. We should also consider that the prevalence of *fibrillar patterns* frequently detected on ANs of the plantar arch, a non-weight-bearing area, in this study, may be biased by the subgroup numerosity.

Concerning the anatomo-functional analysis, the following conclusions were reached:

- The “plantar arch” ended up being the more challenging area for both residents and dermatologists, hosting the more “difficult” and equivocal lesions such as ANs exhibiting *asymmetry of structures, colors*, *irregular blotches* and *irregular diffuse pigmentation*, or EM cases exhibiting *parallel-furrow pattern* and/or more *regression structures* than in other areas.
- When looking at an aMPPL of the *heel* area, *parallel furrow pattern* (*p* = 0.014) or *lattice-like* (*p* = 0.001), patterns should be searched in order to identify benign cases, while *asymmetry of colors* (*p* = 0.002) and *regression structures* (*p* = 0.025) for malignant ones; if an aMPPLs in *plantar arch* exhibits a *lattice-like pattern, it* is likely to be benign (*p* = 0.012), while it is likely to be an EM if one observes either *asymmetry of structures, asymmetry of colors, regression structures,* or *blue-white veil.*
- Then, in the other macro areas, only malignancy-suggestive features were really different (*p* < 0.05) between EMs and ANs, namely in the *toe area*, the *asymmetry of structures, asymmetry of colors, irregular blotches, regression structures,* or *irregular diffuse pigmentation*; in the *eminence of the sole* area, the *asymmetry of colors*, *regression structures,* and *blue-white veil*.

A deep understanding of these dermoscopic variations according to a specific subarea may help dermatologists with different experience levels in dermoscopy to orient their diagnostic suspect in front of the atypical equivocal melanocytic lesions of palms and soles. 

Further studies are required on this specific subset of difficult lesions in the European population to confirm these preliminary data, especially for palmar aMPPLs.

## Figures and Tables

**Table 1:** Characteristics of the case study—the *iDScore palmoplantar testing set*: 471 cases of atypical melanocytic palmoplantar lesions (aMPPLs) collected from 10 European Centers: significance of the comparison according to histologic diagnosis (*p*) is also shown.

LESION DATA	aMPPLs N= 471	MM N = 94	AN N = 377	*p*
	N (%)/Mean ± SD			
Maximum diameter (mm)	8.24 ± 6.3	15.30 ± 9.95	6.49 ± 3.08	<0.001
**PATIENT DATA**				
Age	45.39 ± 18.96	63.97 ± 14.87	40.75 ± 16.94	<0.001
Male	307 (65.2%)	45 (47.9%)	119 (31.6%)	0.004
Female	164 (34.8%)	49 (52.1%)	258 (68.4%)	
ANAMNESTIC DATA/RISK FACTORS *				
Personal/family history of melanoma—1st degree relative	11 (2.3%)	0 (0.0%)	11 (13.6%)	0.520
Presence of >100 common nevi or >10 AN on the body	24 (5.1%)	3 (30%)	21 (25.9%)	1.000
Chronic traumatism of palms	1 (0.2%)	0 (0%)	1 (1.1%)	1.000
Chronic traumatism of soles	10 (2.1%)	0 (0%)	10 (7.2%)	0.601
Patients’ phototype	355 (75.4%)			0.717
II	94 (20%)	19 (29.7%)	75 (25.8%)	
III	248 (52.7%)	44 (68.8%)	204 (70.1%)	
IV	11 (2.3%)	1 (1.6%)	10 (3.4%)	
V	2 (0.4%)	0 (0.0%)	2 (0.7%)	

* Only positive reports are shown.

**Table 2:** Distribution of the case study (471 atypical melanocytic palmoplantar lesions—aMPPLs) according to anatomic, functional, anatomo-functional, and histopathologic classifications. The significance of distribution according to a specific area is also shown (bold).

**Anatomic Classification**	**Histopathologic Classification**			**Functional Classification** (Pressure /chronic traumatism)	**Anatomo-Functional Classification**	**Histopathologic-Functional Classification**		
	**MM**	**AN**	***p***			**MM**	**AN**	***p***
All areas	94	377				94	377	
*8 subareas of the sole*	86 (19.7%)	349 (80.2%)			*4 areas—sole*	17 (18.1%)	56 (14.9%)	0.422
Anterior lateral eminence	2 (2.1)	13 (3.4)	0.746	Intense	Eminence of the sole area *(intense)*			
Anterior medial eminence	12 (12.8)	25 (6.6)	0.055	Intense				
Central eminence	3 (3.2)	18 (4.8)	0.780	Moderate				
Heel	23 (24.5)	34 (9.0)	**<0.001**	Intense	Heel area	23 (24.5%)	34 (9.0%)	**<0.001**
Plantar region	27 (28.7)	180 (47.7)	**0.001**	None	Plantar area	27 (28.7%)	180 (47.7%)	**0.001**
Interdigital spaces	2 (2.1)	25 (6.6)	0.134	None	Toe area *(moderate)*	19 (20.2%)	79 (21%)	1.000
Lateral surface fingers	8 (8.5)	26 (6.9)	0.655	Mild				
Plantar surface fingers	9 (9.6)	28 (7.4)	0.520	Moderate				
*9 Subareas of the palms*	8	28			*3 areas—palms*	8	28	0.392
Fingertips (hand)	2 (2.1)	0 (0.0%)	0.039	Intense	Fingers area (*moderate*)			
Interdigital spaces	0 (0.0%)	3 (0.8)	1.000	Mild		3 (3.2%)	6 (1.6%)	
Lateral surface fingers	1 (1.1%)	0 (0.0%)	0.200	Moderate				
Volar surface fingers	0 (0.0%)	3 (0.8%)	1.000	Intense				
Proximal phalangeal surface	0 (0%)	0 (0%)		intense				
Ipothenar surface	0 (0.0%)	8 (2.1%)	0.367	Moderate	Palmar medial area *(moderate)*	3 (3.2%)	14 (3.7%)	1.000
Metacarpal surface	3 (3.2%)	6 (1.6%)	0.392	Moderate				
Central metacarpal	0 (0.0%)	2 (0.5%)	1.000	Mild	Palmar lateral area *(moderate-mild)*	2 (2.1%)	8 (2.1%)	1.000
Thenar surface	2 (2.1%)	6 (1.6%)	0.663	Moderate				

**Table 3:** Distribution of 11 dermoscopic patterns assessed by 148 participants in 435 plantar atypical melanocytic lesions and variation analysis (*p*) according to specific plantar location and personal dermoscopic skills. Significant values are in bold.

Dermoscopic Pattern Analysis	Skill Levels I–II (n = 87) °					Skill Levels III–IV (n = 61) °				
4 Subareas *	*Eminence Sole*	*Heel*	*Plantar arch*	*Toe*	*p*	*Eminence* *Sole*	*Heel*	*Plantar* *Arch*	*Toe*	*p*
**Melanoma cases (n)**	17	23	27	19		17	23	27	19	
Asymmetry of structures	9 (52.9)	15 (65.2)	21 (77.8)	14 (73.7)	0.342	9 (52.9)	12 (52.2)	22 (81.5)	18 (94.7)	**0.004**
Asymmetry of colors	13 (76.5)	15 (65.2)	25 (92.6)	14 (73.7)	0.124	11 (64.7)	16 (69.6)	23 (85.2)	15 (78.9)	0.391
Parallel ridge pattern	3 (17.6)	3 (13.0)	2 (7.4)	3 (15.8)	0.749	2 (11.8)	8 (34.8)	5 (18.5)	2 (10.5)	0.171
irregular blotches	3 (17.6)	6 (26.1)	8 (29.6)	6 (31.6)	0.786	6 (35.3)	6 (26.1)	9 (33.3)	12 (63.2)	0.080
regression structures	8 (47.1)	8 (34.8)	13 (48.1)	5 (26.3)	0.416	8 (47.1)	9 (39.1)	15 (55.6)	7 (36.8)	0.558
Blue-white veil	5 (29.4)	5 (21.7)	11 (40.7)	7 (36.8)	0.515	8 (47.1)	6 (26.1)	10 (37.0)	6 (31.6)	0.564
Irregular streaks	2 (11.8)	1 (4.3)	4 (14.8)	3 (15.8)	0.621	3 (17.6)	3 (13.0)	6 (22.2)	3 (15.8)	0.856
Irregular diffuse Pigmentation	5 (29.4)	5 (21.7)	3 (11.1)	5 (26.3)	0.448	4 (23.5)	3 (13.0)	7 (25.9)	5 (26.3)	0.673
Irregular fibrillar pattern	0 (0.0)	1 (4.3)	1 (3.7)	0 (0.0)	0.683	3 (17.6)	1 (4.3)	3 (11.1)	2 (10.5)	0.601
Parallel furrow pattern	1 (5.9)	1 (4.3)	0 (0.0)	0 (0.0)	0.483	4 (23.5)	0 (0.0)	0 (0.0)	4 (21.1)	**0.006**
Regular fibrillar pattern	2 (11.8)	2 (8.7)	0 (0.0)	1 (5.3)	0.373	0 (0.0)	1 (4.3)	0 (0.0)	2 (10.5)	0.218
Lattice-like pattern	0 (0.0)	0 (0.0)	0 (0.0)	0 (0.0)	-	1 (5.9)	0 (0.0)	1 (3.7)	1 (5.3)	0.729
**Nevi cases (n)**	55	32	179	77		50	31	165	68	
Asymmetry of structures	17 (30.9)	3 (9.4)	44 (24.6)	21 (27.3)	0.142	16 (32.0)	9 (29.0)	45 (27.3)	22 (32.4)	0.847
Asymmetry of colors	15 (27.3)	4 (12.5)	52 (29.1)	23 (29.9)	0.256	15 (30.0)	7 (22.6)	45 (27.3)	22 (32.4)	0.752
Parallel ridge pattern	4 (7.3)	2 (6.2)	10 (5.6)	10 (13.0)	0.229	7 (14.0)	3 (9.7)	15 (9.1)	9 (13.2)	0.685
Irregular blotches	6 (10.9)	2 (6.2)	15 (8.4)	7 (9.1)	0.893	13 (26.0)	3 (9.7)	24 (14.5)	14 (20.6)	0.150
Regression structures	6 (10.9)	1 (3.1)	19 (10.6)	8 (10.4)	0.608	5 (10.0)	3 (9.7)	21 (12.7)	5 (7.4)	0.672
Blue-white veil	8 (14.5)	1 (3.1)	14 (7.8)	11 (14.3)	0.139	4 (8.0)	2 (6.5)	12 (7.3)	8 (11.8)	0.694
Irregular streaks	9 (16.4)	1 (3.1)	6 (3.4)	8 (10.4)	**0.004**	4 (8.0)	2 (6.5)	19 (11.5)	9 (13.2)	0.678
Irregular diffuse Pigmentation	4 (7.3)	0 (0.0)	16 (8.9)	6 (7.8)	0.375	3 (6.0)	3 (9.7)	16 (9.7)	1 (1.5)	0.159
Irregular fibrillar pattern	7 (12.7)	2 (6.2)	17 (9.5)	0 (0.0)	**0.024**	4 (8.0)	4 (12.9)	21 (12.7)	8 (11.8)	0.833
Parallel furrow pattern	12 (21.8)	9 (28.1)	50 (27.9)	23 (29.9)	0.769	20 (40.0)	9 (29.0)	37 (22.4)	26 (38.2)	**0.027**
Regular fibrillar pattern	5 (9.1)	8 (25.0)	25 (14.0)	4 (5.2)	**0.024**	9 (18.0)	7 (22.6)	17 (10.3)	5 (7.4)	0.079
Lattice-like pattern	7 (12.7)	11 (34.4)	38 (21.2)	14 (18.2)	0.105	9 (18.0)	13 (41.9)	47 (28.5)	13 (19.1)	**0.047**

* Toes area (plantar surface of the fingers + lateral surface of the fingers + interdigital spaces); eminence of the sole area (anterior eminence + central eminence + antero-medial eminence); plantar arch area; heel. ° Skill levels I–II: up to 4 years-experience in dermoscopy; skill levels III–IV: ≥5 years-experience in dermoscopy.

**Table 4:** Distribution of 11 dermoscopic patterns assessed by 56 participants in 36 palmar atypical melanocytic lesions and variation analysis (*p*) according to palmar location and personal dermoscopic skills.

Dermoscopic Pattern Analysis	Skill Levels I–II (n = 26) °			Skill Levels III–IV (n = 30) °		
3 Subareas *	*Fingers*	*Palmar Lateral*	*Palmar* *Medial*	*Fingers*	*Palmar* *Lateral*	*Palmar* *Medial*
**Melanoma cases (n)**	3	3	2	3	3	2
Asymmetry of structures	2 (66.7)	2 (66.7)	2 (100.0)	2 (66.7)	2 (66.7)	1 (50.0)
Asymmetry of colors	3 (100.0)	2 (66.7)	2 (100.0)	3 (100.0)	2 (66.7)	1 (50.0)
Parallel ridge pattern	0 (0.0)	0 (0.0)	0 (0.0)	3 (100.0)	0 (0.0)	0 (0.0)
Irregular blotches	1 (33.3)	2 (66.7)	1 (50.0)	2 (66.7)	1 (33.3)	0 (0.0)
Regression structures	1 (33.3)	1 (33.3)	1 (50.0)	2 (66.7)	2 (66.7)	0 (0.0)
Blue-white veil	1 (33.3)	1 (33.3)	0 (0.0)	1 (33.3)	1 (33.3)	0 (0.0)
Irregular streaks	0 (0.0)	0 (0.0)	0 (0.0)	0 (0.0)	0 (0.0)	0 (0.0)
Irregular diffuse pigmentation	2 (66.7)	1 (33.3)	1 (50.0)	1 (33.3)	1 (33.3)	0 (0.0)
Irregular fibrillar pattern	1 (33.3)	0 (0.0)	0 (0.0)	0 (0.0)	0 (0.0)	0 (0.0)
Parallel furrow pattern	1 (33.3)	0 (0.0)	0 (0.0)	1 (33.3)	0 (0.0)	0 (0.0)
Regular fibrillar pattern	0 (0.0)	0 (0.0)	0 (0.0)	0 (0.0)	0 (0.0)	0 (0.0)
Lattice-like pattern	0 (0.0)	1 (33.3)	0 (0.0)	0 (0.0)	1 (33.3)	0 (0.0)
**Nevi cases (n)**	6	14	8	6	12	8
Asymmetry of structures	0 (0.0)	6 (42.9)	4 (50.0)	1 (16.7)	4 (33.3)	1 (12.5)
Asymmetry of colors	0 (0.0)	5 (35.7)	2 (25.0)	1 (16.7)	5 (41.7)	2 (25.0)
Parallel ridge pattern	0 (0.0)	1 (7.1)	1 (12.5)	4 (66.7)	0 (0.0)	0 (0.0)
Irregular blotches	0 (0.0)	1 (7.1)	1 (12.5)	2 (33.3)	0 (0.0)	1 (12.5)
Regression structures	0 (0.0)	0 (0.0)	0 (0.0)	0 (0.0)	1 (8.3)	0 (0.0)
Blue-white veil	0 (0.0)	0 (0.0)	1 (12.5)	1 (16.7)	0 (0.0)	0 (0.0)
Irregular streaks	1 (16.7)	2 (14.3)	1 (12.5)	0 (0.0)	0 (0.0)	2 (25.0)
Irregular diffuse pigmentation	1 (16.7)	0 (0.0)	0 (0.0)	1 (16.7)	1 (8.3)	0 (0.0)
Irregular fibrillar pattern	1 (16.7)	1 (7.1)	0 (0.0)	0 (0.0)	0 (0.0)	1 (12.5)
Parallel furrow pattern	2 (33.3)	5 (35.7)	5 (62.5)	2 (33.3)	7 (58.3)	4 (50.0)
Regular fibrillar pattern	0 (0.0)	1 (7.1)	2 (25.0)	0 (0.0)	0 (0.0)	0 (0.0)
Lattice-like pattern	2 (33.3)	5 (35.7)	1 (12.5)	1 (16.7)	2 (16.7)	3 (37.5)

* Fingers (fingertips + lateral surface of the fingers + interdigital spaces + volar surface of the finger + proximal phalangeal surface); palmar lateral (metacarpal area + hypothenar); palmar medial: thenar + central metacarpal. ° Skill levels I–II: up to 4 years-experience in dermoscopy; skill levels III–IV: ≥5 years-experience in dermoscopy.

**Table 5:** Distribution analysis (*p*) according to histology and plantar location among 4 plantar areas of 12 dermoscopic patterns assessed in tele-dermoscopy by 61 dermatologists (i.e., ≥5-year experience in dermatology) in 400 atypical melanocytic plantar lesions. Significant values are in bold.

4 Subareas	Eminence of the Sole *			Heel			Toe °			Plantar Arch		
N (%)/Mean ± SD	MM	AN	*p*	MM	AN	*p*	MM	AN	*p*	MM	AN	*p*
**Dermoscopic patterns**	17	50		23	31		19	68		27	165	
Asymmetry of structures	9 (52.9)	16 (32.0)	0.211	12 (52.2)	9 (29.0)	0.149	18 (94.7)	22 (32.4)	**<0.001**	22 (81.5)	45 (27.3)	**<0.001**
Asymmetry of colors	11 (64.7)	15 (30.0)	**0.025**	16 (69.6)	7 (22.6)	**0.002**	15 (78.9)	22 (32.4)	**0.001**	23 (85.2)	45 (27.3)	**0.001**
Parallel ridge pattern	2 (11.8)	7 (14.0)	1.000	8 (34.8)	3 (9.7)	0.054	2 (10.5)	9 (13.2)	1.000	5 (18.5)	15 (9.1)	0.251
Irregular blotches	6 (35.3)	13 (26.0)	0.672	6 (26.1)	3 (9.7)	0.218	12 (63.2)	14 (20.6)	**0.001**	9 (33.3)	24 (14.5)	0.034
Regression structures	8 (47.1)	5 (10.0)	**0.003**	9 (39.1)	3 (9.7)	**0.025**	7 (36.8)	5 (7.4)	**0.004**	15 (55.6)	21 (12.7)	**<0.001**
Blue-white veil	8 (47.1)	4 (8.0)	**0.001**	6 (26.1)	2 (6.5)	0.105	6 (31.6)	8 (11.8)	0.085	10 (37.0)	12 (7.3)	**<0.001**
Irregular streaks	3 (17.6)	4 (8.0)	0.506	3 (13.0)	2 (6.5)	0.725	3 (15.8)	9 (13.2)	1.000	6 (22.2)	19 (11.5)	0.221
Irregular diffuse Pigmentation	4 (23.5)	3 (6.0)	0.114	3 (13.0)	3 (13.3)	1.000	5 (26.3)	1 (1.5)	**0.001**	7 (25.9)	16 (9.7)	0.037
Irregular fibrillar pattern	3 (17.6)	4 (8.0)	0.506	1 (4.3)	4 (12.9)	0.550	2 (10.5)	8 (11.8)	1.000	3 (11.1)	21 (12.7)	1.000
Parallel furrow pattern	4 (23.5)	20 (40.0)	0.352	0 (0.0)	9 (29.0)	**0.014**	4 (21.1)	26 (38.2)	0.263	0 (0.0)	37 (22.4)	0.013
Regular fibrillar pattern	0 (0.0)	9 (18.0)	0.142	1 (4.3)	7 (22.6)	0.140	2 (10.5)	5 (7.4)	1.000	0 (0.0)	17 (10.3)	0.167
Lattice-like pattern	1 (5.9)	9 (18.0)	0.414	0 (0.0)	13 (41.9)	**0.001**	1 (5.3)	13 (19.1)	0.271	1 (3.7)	47 (28.5)	**0.012**

* Eminence of the sole area: anterior eminence + central eminence + antero-medial eminence. ° Toes area: plantar surface of the fingers + lateral surface of the fingers + interdigital spaces.

**Table 6:** Distribution of 12 dermoscopic patterns assessed in tele-dermoscopy by 43 dermatologists (i.e., ≥5-year experience in dermatology) according to histology and palmar location among 3 areas, in 34 atypical melanocytic skin lesions.

PALMAR AREAS *	Fingers		Palmar Lateral		Palmar Medial	
N (%)/Mean ± SD	MM	AN	MM	AN	MM	AN
**Dermoscopic patterns**	3	6	3	12	2	8
Asymmetry of structures	2 (66.7)	1 (16.7)	2 (66.7)	4 (33.3)	1 (50.0)	1 (12.5)
Asymmetry of colours	3 (100.0)	1 (16.7)	2 (66.7)	5 (41.7)	1 (50.0)	2 (25.0)
Parallel ridge pattern	3 (100.0)	4 (66.7)	0 (0.0)	0 (0.0)	0 (0.0)	0 (0.0)
Irregular blotches	2 (66.7)	2 (33.3)	1 (33.3)	0 (0.0)	0 (0.0)	1 (12.5)
Regression structures	2 (66.7)	0 (0.0)	2 (66.7)	1 (8.3)	0 (0.0)	0 (0.0)
Blue-white veil	1 (33.3)	1 (16.7)	1 (33.3)	0 (0.0)	0 (0.0)	0 (0.0)
Irregular streaks	0 (0.0)	0 (0.0)	0 (0.0)	0 (0.0)	0 (0.0)	2 (25.0)
Irregular diffuse pigmentation	1 (33.3)	1 (16.7)	1 (33.3)	1 (8.3)	0 (0.0)	0 (0.0)
Irregular fibrillar pattern	0 (0.0)	0 (0.0)	0 (0.0)	0 (0.0)	0 (0.0)	1 (12.5)
Parallel furrow pattern	1 (33.3)	2 (33.3)	0 (0.0)	7 (58.3)	0 (0.0)	4 (50.0)
Regular fibrillar pattern	0 (0.0)	0 (0.0)	0 (0.0)	0 (0.0)	0 (0.0)	0 (0.0)
Lattice-like pattern	0 (0.0)	1 (16.7)	1 (33.3)	2 (16.7)	0 (0.0)	3 (37.5)

* Finger area (fingertips + lateral surface of the fingers + interdigital spaces + volar surface of the finger + proximal phalangeal surface); palmar lateral area (metacarpal area + hypothenar); palmar medial: thenar + central metacarpal.

## Data Availability

Data are available from the corresponding author upon reasonable request.
