# Correction: Tognetti et al. Pattern Analysis of Benign and Malignant Atypical Melanocytic Skin Lesions of Palms and Soles: Variations of Dermoscopic Features According to Anatomic Site and Personal Experience. *Life* 2024, *14*, 659

**DOI:** 10.3390/life14121687

**Published:** 2024-12-20

**Authors:** Linda Tognetti, Alessandra Cartocci, Elvira Moscarella, Aimilios Lallas, Emi Dika, Maria Concetta Fargnoli, Caterina Longo, Gianluca Nazzaro, John Paoli, Ignazio Stanganelli, Serena Magi, Francesco Lacarrubba, Paolo Broganelli, Jean-Luc Perrot, Mariano Suppa, Harald Kittler, Roberta Giuffrida, Elisa Cinotti, Lo Conte Sofia, Gennaro Cataldo, Gabriele Cevenini, Pietro Rubegni

**Affiliations:** 1 Dermatology Unit, Department of Medical, Surgical and Neurosciences, University of Siena, 53100 Siena, Italy; 2 Bioengineering and Biomedical Data Science Lab, Department of Medical Biotechnologies, University of Siena, 53100 Siena, Italy; 3 Dermatology Unit, University of Campania Luigi Vanvitelli, 81100 Naples, Italy; 4 First Department of Dermatology, Aristotle University, 54124 Thessaloniki, Greece; 5 Oncologic Dermatology Unit, IRCCS Azienda Ospedaliero-Universitaria di Bologna, 40138 Bologna, Italy; 6 Department of Medical and Surgical Sciences (DIMEC), Alma Mater Studiorum University of Bologna, 40138 Bologna, Italy; 7 Dermatology Unit, University of L’Aquila, 67100 L’Aquila, Italy; 8 Department of Dermatology, University of Modena and Reggio Emilia, 41125 Modena, Italy; 9 Skin Cancer Center, Azienda Unità Sanitaria Locale-IRCCS di Reggio Emilia, 42123 Reggio Emilia, Italy; 10 Fondazione IRCCS Ca’ Granda Ospedale Maggiore Policlinico, 20122 Milan, Italy; 11 Department of Dermatology and Venereology, Institute of Clinical Sciences, Sahlgrenska Academy, University of Gothenburg, 41390 Gothenburg, Sweden; 12 Department of Dermatology and Venereology, Region Västra Götaland, Sahlgrenska University Hospital, 41345 Gothenburg, Sweden; 13 Skin Cancer Unit, Scientific Institute of Romagna for the Study of Cancer, IRCCS, IRST, 47014 Meldola, Italy; 14 Department of Dermatology, University of Parma, 43121 Parma, Italy; 15 Dermatology Clinic, University of Catania, 95123 Catania, Italy; 16 Dermatology Unit, University Hospital of Torino, 10126 Torino, Italy; 17 Dermatology Unit, University Hospital of St-Etienne, 42270 Saint Etienne, France; 18 Department of Dermatology, Hôpital Erasme, Université Libre de Bruxelles, 1070 Brussels, Belgium; 19 Department of Dermatology, Institut Jules Bordet, Université Libre de Bruxelles, 1070 Brussels, Belgium; 20 Groupe d’Imagerie Cutanée Non Invasive (GICNI) of the Société Française de Dermatologie (SFD), 75008 Paris, France; 21 Department of Dermatology, Medical University of Vienna, 1090 Vienna, Austria; 22 Department of Clinical and Experimental Medicine, Dermatology, University of Messina, 98122 Messina, Italy

## Addition of One Author

“Harald Kittler” was not included as an author in the original publication [1]. The corrected affiliation and Author Contributions statement appears here.

21 Department of Dermatology, Medical University of Vienna, 1090 Vienna, Austria

**Author Contributions:** Conceptualization, L.T. and P.R.; methodology, L.T., P.R., A.C. and G.C. (Gabriele Cevenini); software, G.C. (Gabriele Cevenini) and G.C. (Gennaro Cataldo); formal analysis, A.C. and L.C.S.; investigation, L.T., A.C., E.M., E.D., A.L., I.S., S.M., G.N., J.P., M.C.F., C.L., P.B., M.S., F.L., H.K. and J.-L.P.; resources, E.M., E.D., A.L., I.S., S.M., G.N., J.P., M.C.F., C.L., P.B., M.S., F.L., E.C., H.K. and J.-L.P.; data curation, L.T., A.C. and L.C.S.; writing—original draft preparation, L.T. and A.C.; writing—review and editing, R.G., E.M. and P.R.; supervision, G.C. (Gabriele Cevenini), E.C. and P.R.; project administration, L.T. and A.C. All authors have read and agreed to the published version of the manuscript.

The authors state that the scientific conclusions are unaffected. This correction was approved by the Academic Editor. The original publication has also been updated.
